# Glioblastoma Distance From the Subventricular Neural Stem Cell Niche Does Not Correlate With Survival

**DOI:** 10.3389/fonc.2020.564889

**Published:** 2020-12-11

**Authors:** Akshitkumar M. Mistry, Nishit Mummareddy, Sanjana Salwi, Larry T. Davis, Rebecca A. Ihrie

**Affiliations:** ^1^ Department of Neurological Surgery, Vanderbilt University Medical Center, Nashville, TN, United States; ^2^ School of Medicine, Vanderbilt University, Nashville, TN, United States; ^3^ Department of Radiology, Vanderbilt University Medical Center, Nashville, TN, United States; ^4^ Department of Cell and Developmental Biology, Vanderbilt University, Nashville, TN, United States

**Keywords:** glioblastoma, subventricular zone, survival, stem cell, glioma

## Abstract

**Objective:**

To determine the relationship between survival and glioblastoma distance from the ventricular-subventricular neural stem cell niche (VSVZ).

**Methods:**

502 pre-operative gadolinium-enhanced, T1-weighted MRIs with glioblastoma retrieved from an institutional dataset (n = 252) and The Cancer Imaging Atlas (n=250) were independently reviewed. The shortest distance from the tumor contrast enhancement to the nearest lateral ventricular wall, the location of the VSVZ, was measured (GBM-VSVZ_Dist_). The relationship of GBM-VSVZ_Dist_ with the proportion of glioblastomas at each distance point and overall survival was explored with a Pearson’s correlation and Cox regression model, respectively, adjusting for the well-established glioblastoma prognosticators.

**Results:**

244/502 glioblastomas had VSVZ contact. The proportion of non-VSVZ-contacting glioblastomas correlated inversely with GBM-VSVZ_Dist_ (partial Pearson’s correlation adjusted for tumor volume R=-0.79, p=7.11x10^-7^). A fit of the Cox regression model adjusted for age at diagnosis, Karnofsky performance status score, post-operative treatment with temozolomide and/or radiotherapy, *IDH1/2* mutation status, *MGMT* promoter methylation status, tumor volume, and extent of resection demonstrated a significantly decreased overall survival only when glioblastoma contacted the VSVZ. Overall survival did not correlate with GBM-VSVZ_Dist_.

**Conclusions:**

In the two independent cohorts analyzed, glioblastomas at diagnosis were found in close proximity or in contact with the VSVZ with a proportion that decreased linearly with GBM-VSVZ_Dist_. Patient survival was only influenced by the presence or absence of a gadolinium-enhanced glioblastoma contact with the VSVZ. These results may guide analyses to test differential effectiveness of VSVZ radiation in VSVZ-contacting and non-contacting glioblastomas and/or inform patient selection criteria in clinical trials of glioblastoma radiation.

## Introduction

The median survival of patients with glioblastoma is 16 months (1, 2). Survival is even lower in those patients in whom the glioblastoma has invaded or contacted the ventricular-subventricular zone (VSVZ) at diagnosis (3, 4). The VSVZ is a neural stem cell niche in the lateral walls of the lateral ventricles in the brain (5–7). Recent results have supported the hypothesis that VSVZ houses the cellular origins of some human glioblastomas (7, 8).

Glioblastomas with and without VSVZ invasion or contact are not genomically distinct in bulk profiling (9, 10). Therefore, the more severe clinical phenotype of those patients is likely due to the microenvironment of the VSVZ (9). The microenvironment is especially attractive to glioblastoma cells (11, 12), and factors released by the VSVZ can mediate resistance to radiation therapy in glioblastoma cells (13). Consistent with these reports, recurrence is earlier in those patients in whom the initial glioblastoma invaded or contacted the VSVZ (3, 4).

Research efforts in this area have been conducted by dichotomizing glioblastomas as having VSVZ contact or not, demonstrating survival is lower with contact. Whether clinical outcome is related continuously with glioblastoma distance from the VSVZ (GBM-VSVZ_Dist_) is unknown. Therefore, in this brief report, we conducted survival analyses to uncover the relationship of patient survival with glioblastoma distance from the VSVZ that yielded consistent findings in two distinct datasets.

## Methods

**Figure 1:**
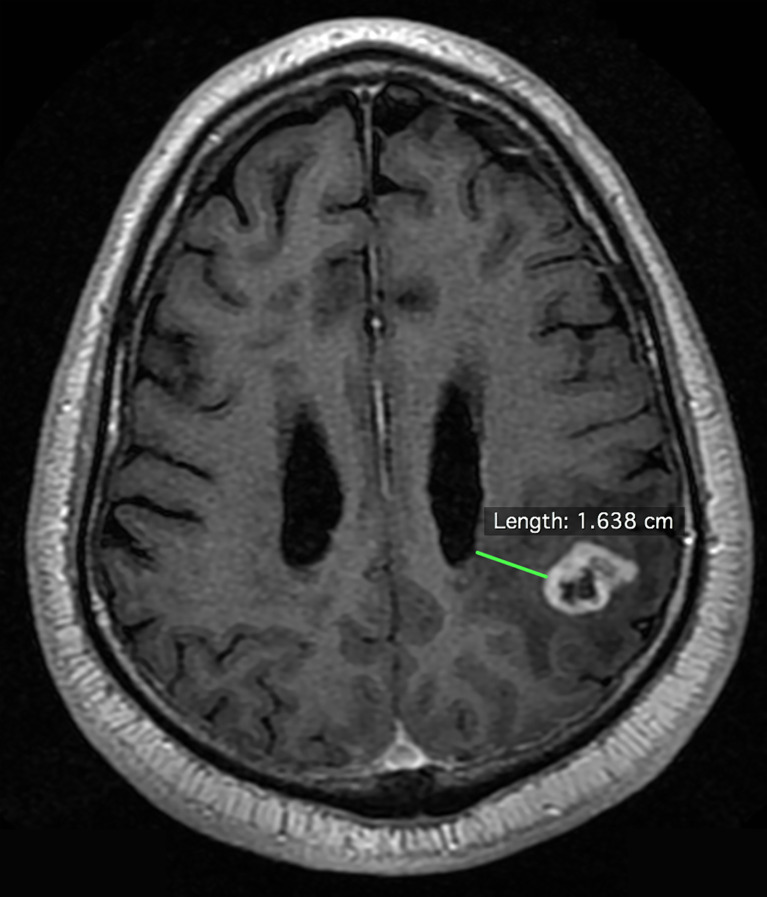
Example measurement of the shortest distance between the enhancing edge of a glioblastoma to the lateral wall of the lateral ventricle on an axial pre-operative gadolinium-enhanced, T1-weighted magnetic resonance image.

A total of 502 patients with histologically confirmed glioblastoma were analyzed: 252 from a prospectively maintained single-institution (VUMC) registry and 250 from The Cancer Imaging Atlas (TCIA) (14). In the institutional dataset, the median year of diagnosis was 2012 [interquartile range: 2009-2015] and the patients were followed to 2017. Institutional review board approval was obtained; patient consent was waived. Two independent, outcome-blinded reviews of pre-operative gadolinium-enhanced, T1-weighted MRIs were conducted. One of these assessments was made by a board-certified neuro-radiologist. All institutional MRIs were obtained using a 1.5 or 3-tesla magnetic field, and details of the MRIs in the TCIA are published (14). As per standard assessment, all MRIs were first dichotomized as demonstrating contact of the tumor contrast enhancement with the lateral ventricular ependyma—the location of the VSVZ—or not. GBM-VSVZ_Dist_ was then measured for all non-VSVZ-contacting glioblastomas. Specifically, the shortest distance from the tumor contrast enhancement to the nearest lateral ventricular wall visible on any one of three MRI dimensions (axial, coronal, or sagittal) was measured to the nearest millimeter (mm) (**Figure 1**). All disagreements were resolved by group consensus. Glioblastoma volume contained within contrast enhancement was also calculated. All of these radiological assessments were performed using OsiriX Lite software (version 9.4, Pixmeo, Geneva, Switzerland).

Corresponding clinical data were obtained from the institution registry and The Cancer Genome Atlas (9). Patient survival was recorded as the time from initial diagnosis to death or to the last time known to be alive (censored survival). The relationship between GBM-VSVZ_Dist_ and survival was assessed using the Cox regression model, adjusting for well-established glioblastoma prognosticators. These included age at diagnosis (treated as a continuous variable), pre-operative Karnofsky performance status score (KPS; categorized into 100-80, 70-50, 40-0), postoperative treatment with temozolomide and/or radiotherapy (yes or no), *IDH1/2* mutation status (wildtype, mutant, or unassessed), *MGMT* promoter methylation status (methylated, unmethylated, or unassessed), tumor volume (continuous variable), and extent of resection of the enhancing portion of the glioblastoma. The extent of resection was categorized as biopsy (<50%), subtotal (50%–95%), near-total (95%–99%) and gross total (100%) based on independent assessments by a neurosurgeon and neuroradiologist of the postoperative, post-contrast MRI obtained within 24 h after the operation. The adjusted Cox models were confirmed to meet the proportional hazards assumption, which was tested by assessing the significance of the relationship between Schoenfeld residuals and time for the overall model. The results of the Cox models are reported as hazard ratios with 95% confidence intervals. Finally, the relationship between GBM-VSVZ_Dist_ and adjusted hazard ratios were depicted using a quadratic spline fit.

Standard descriptive statistical methods were used to report variables and compare distributions of continuous and proportions of categorical variables. Correlation between two variables was assessed using Pearson’s correlation treating both variables continuously. Statistical significance was claimed with a two-sided p-value of ≤ 0.05 or 95% confidence intervals that did not span 1. All analyses were conducted using R version 3.4 (R Foundation for Statistical Computing, Vienna, Austria) and the Survminer package (Version 0.4.0).

## Results

**Figure 2:**
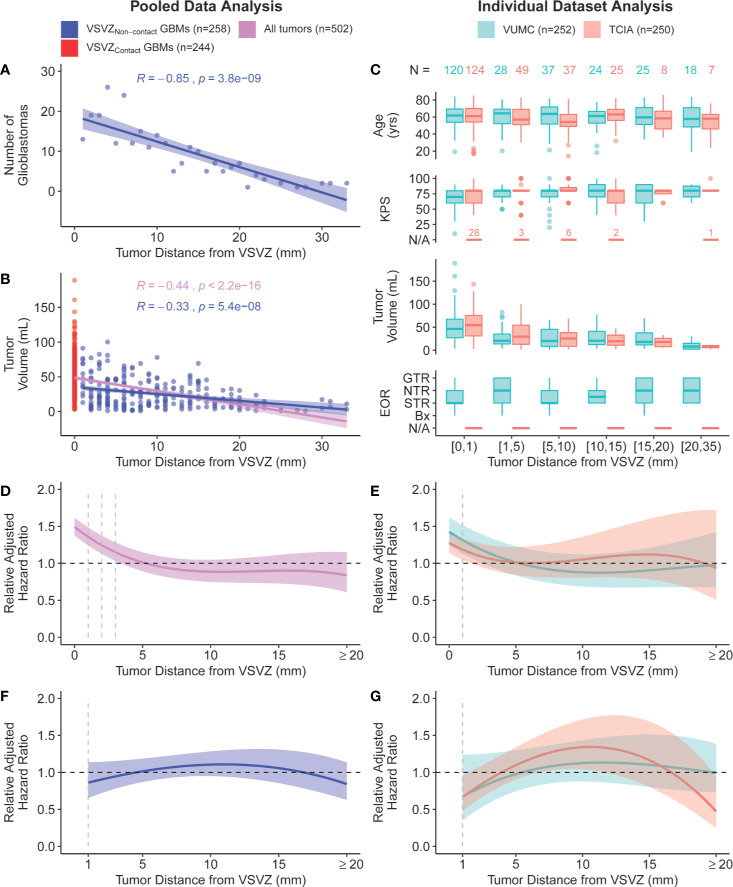
Scatter plot of **(A)** the number of non-VSVZ-contacting glioblastomas (VSVZ_Non-contact_ GBMs) and **(B)** tumor volume plotted in relation to their distance to the VSVZ (GBM-VSVZ_Dist_). The relationships are quantified with a Pearson’s correlation. **(C)** Boxplot distributions of age, Karnofsky performance status score (KPS), glioblastoma volume, and extent of resection of enhancing glioblastoma [EOR; <50% (biopsy, bx); 50%–95% (subtotal, STR); 95%–99% (near total, NTR); 100% (gross total, GTR)] plotted against GBM-VSVZ_Dist_ grouped into select ranges in the single-institution (orange) and The Cancer Imaging Archive (TCIA; teal) datasets. The relationship between GBM-VSVZ_Dist_ and adjusted hazard ratio relative to the hazard at GBM-VSVZ_Dist_ of 5mm in **(D)** pooled data and **(E)** within each dataset is depicted using a quadratic spline fit. The size of the colored ribbon represents the 95% confidence interval. The hazard ratio is adjusted for the following: age at diagnosis (continuous), Karnofsky performance status score (KPS: 100-80, 70-50, 40-0, or not available, as in the case of few TCIA patients denoted in **(C)**, post-operative treatment with temozolomide and/or radiotherapy (yes or no), *IDH1/2* mutation status (wildtype, mutant, or unassessed), *MGMT* promoter methylation status (methylated, unmethylated, or unassessed), tumor volume, and EOR (not available in TCIA hence unadjusted in the TCIA and pooled analyses). **(F, G)** Prior analyses repeated with only non-VSVZ-contacting glioblastomas.

Patient and glioblastoma characteristics are listed in **Table 1**. Out of 502 glioblastomas, 244 (48.6%) had VSVZ contact (GBM-VSVZ_Dist_=0). Interestingly, the number of non-VSVZ-contacting glioblastomas correlated inversely with GBM-VSVZ_Dist_ (R=-0.85, p=3.8x10^-9^; **Figure 2A**). This correlation was partly confounded by the inverse correlation of tumor volume with GBM-VSVZ_Dist_ (R=-0.44, p=2.2x10^-16^; **Figure 2B**); therefore, it was adjusted for the median tumor volume at each GBM-VSVZ_Dist_ value and remained significant (partial Pearson’s correlation R=-0.79, p=7.11x10^-7^). KPS and extent of resection significantly correlated with GBM-VSVZ_Dist_ in the institutional VUMC dataset (R=0.19, p=0.002; R=0.28, p=5.7x10^-6^, respectively), but not in the TCIA dataset (in which extent of resection data are not available; **Figure 2C**).

**Table 1:** Patient and tumor characteristics of the two cohorts analyzed.

	VUMC (n=252)	TCIA (n=250)	P-value
VSVZ-contacting (%)	120 (47.6)	124 (49.6)	0.723
Age [years; median (IQR)]	61.63 [51.93, 69.44]	59.50 [52.00, 69.00]	0.324
Tumor volume [median (IQR)]	30.20 [14.84, 51.77]	33.09 [17.77, 60.59]	0.082
KPS (%)			<0.001
100-80	123 (48.8)	157 (62.8)	
70-50	111 (44.0)	43 (17.2)	
40-10	18 (7.1)	10 (4.0)	
Unknown	0 (0.0)	40 (16.0)	
*IDH1/2* mutation status (%)			0.001
WT	222 (88.1)	189 (75.6)	
Mutant	8 (3.2)	12 (4.8)	
Unknown	22 (8.7)	49 (19.6)	
*MGMT* promoter methylation status (%)			<0.001
Unmethylated	166 (65.9)	82 (32.8)	
Methylated	55 (21.8)	82 (32.8)	
Unknown	31 (12.3)	86 (34.4)	
Temozolomide (%)	202 (80.2)	165 (66.0)	0.001
Radiotherapy (%)	223 (88.5)	188 (75.2)	<0.001
Extent of Resection (%)			
Biopsy	17 (6.7)	N/A	
STR	126 (50.0)	N/A	
NTR	64 (25.4)	N/A	
GTR	45 (17.9)	N/A	

Next, we conducted survival analyses by fitting a Cox regression model, adjusting for the available co-variables (**Table 1**). The adjusted fit demonstrated a significantly decreased survival, or increased hazard, when glioblastoma contacted the VSVZ relative to the hazard at GBM-VSVZ_Dist_ of 5mm (**Figures 2D, E**). An increased hazard was also noted within the immediate proximity (≤1–3 mm) of VSVZ. However, it was concluded to be an artifact of the fit manifested by the greatly increased hazard associated with VSVZ contact, because in an adjusted Cox regression analysis of only the non-VSVZ-contacting glioblastomas, the hazard ratio remained non-significantly altered along the range of GBM-VSVZ_Dist_ values (**Figures 2F, G**).

## Discussion

Recent studies in mouse models and human intraoperative glioblastoma samples suggest that genetically altered neural stem cells can migrate out of the VSVZ and ultimately generate glioblastomas (8). Select glioblastoma cells can, in turn, be attracted to the VSVZ (12, 13). Complimenting these discoveries, our results revealed that about half of glioblastomas radiographically contact the VSVZ at diagnosis. The remaining tumors were found near the VSVZ with a frequency that decreased linearly with GBM-VSVZ_Dist_.

The decreasing number of patients with greater GBM-VSVZ_Dist_ added a limitation for the analysis, as it resulted in increasing confidence intervals around the hazard ratio when examining individual datasets (**Figure 2E**). Hence, we pooled the datasets to address this limitation, yielding a more constant and precise range of confidence interval around the hazard ratio, which remained steady around 1 (**Figures 2D, F**). Our results are in accordance with MRI probabilistic maps of glioblastoma that highlight areas associated with lower survival (15, 16).

Patient survival was only influenced by the presence or absence of a gadolinium-enhanced glioblastoma contact with the VSVZ (3). Glioblastoma volume, depth, and any potential influence of these variables on the extent of resection may not solely explain the lower survival associated with VSVZ-contacting glioblastomas.

Several studies have sought to understand the molecular basis for the increased malignancy of glioblastomas with VSVZ contact (17–19). Two of them demonstrate a correlation with increased glioblastoma expression of CD133, a glioma stem cell marker, with proximity to the VSVZ (20, 21). However, large bulk tissue analyses have not revealed a consistent molecular signature of VSVZ-contacting glioblastomas (9, 10). Therefore, the role of the microenvironment of the VSVZ is also being probed to understand the increased malignancy of glioblastomas with VSVZ contact. Changes in the disease course that can occur once glioblastoma cells invade the VSVZ, whereupon they are theorized to become therapy-resistant (13), drive recurrence, and disseminate further (22), are hypothesized to explain the lower survival associated with VSVZ-contacting glioblastomas.

This work has some limitations. First, we solely used uniaxial T1-weighted MRIs that did not uniformly include 3D MRI-based sequences, which could lead to biased conclusions. Second, we acknowledge that the enhancing edge of glioblastoma may not represent the true edge of glioblastoma. Therefore, it is critical to rely on a proper combination of MRI studies, and additional histological validation studies are required.

There is a heightened interest in assessing the effectiveness of VSVZ radiation in addition to the standard of care treatment for glioblastoma. For example, one randomized trial is underway (ClinicalTrials.gov Identifier: NCT02177578). Our results may guide analyses of such trials. For example, in existing trials, it may be beneficial to test the differential effectiveness of VSVZ radiation in niche-contacting and non-contacting glioblastomas. Our results may also be used to inform patient selection criteria of future trials in this area; for example, a trial of VSVZ radiation focused only on patients with niche-contacting glioblastoma.

## Data Availability Statement

The raw data supporting the conclusions of this article will be made available by the authors, without undue reservation.

## Ethics Statement

The studies involving human participants were reviewed and approved by Vanderbilt University Medical Center. Written informed consent for participation was not required for this study in accordance with the national legislation and the institutional requirements.

## Author Contributions

AM conceptualized and designed the study, acquired the data, analyzed the data, drafted the manuscript, and revised the manuscript for intellectual content. NM acquired and analyzed the data, and drafted the manuscript. SS acquired the data. LD acquired and interpreted the data. RI conceptualized the study, interpreted the data, and revised the manuscript for intellectual content. All authors contributed to the article and approved the submitted version.

## Funding

National Cancer Institute, National Institutes of Health (F32 CA224962 to A.M.M.), 2018 Burroughs Wellcome Fund Physician-Scientist Institutional Award (1018894 to Vanderbilt University and A.M.M.), National Institute of Neurological Disorders and Stroke (R01 NS 096238 to R.A.I.), Michael David Greene Brain Cancer Fund (R.A.I.), and Vanderbilt-Ingram Cancer Center Ambassadors Award (R.A.I.).

## Conflict of Interest

The authors declare that the research was conducted in the absence of any commercial or financial relationships that could be construed as a potential conflict of interest.
